# The common characteristics and outcomes of multidisciplinary collaboration in primary health care: a systematic literature review

**DOI:** 10.5334/ijic.1359

**Published:** 2015-06-24

**Authors:** Sanneke Schepman, Johan Hansen, Iris D. de Putter, Ronald S. Batenburg, Dinny H. de Bakker

**Affiliations:** NIVEL, Netherlands Institute for Health Services Research, Utrecht, The Netherlands; NIVEL, Netherlands Institute for Health Services Research, Utrecht, The Netherlands; NIVEL, Netherlands Institute for Health Services Research, Utrecht, The Netherlands; NIVEL, Netherlands Institute for Health Services Research, Utrecht, The Netherlands; Information and Organization, Institute of Information and Computing Sciences, Utrecht University, Utrecht, The Netherlands; NIVEL, Netherlands Institute for Health Services Research, Utrecht, The Netherlands; Structure and Organization of Primary Health Care, Tilburg University, Tilburg, The Netherlands

**Keywords:** primary health care, inter-professional relations, multidisciplinary collaboration, integrated care, outcomes

## Abstract

**Introduction:**

Research on collaboration in primary care focuses on specific diseases or types of collaboration. We investigate the effects of such collaboration by bringing together the results of scientific studies.

**Theory and methods:**

We conducted a systematic literature review of PubMed, CINAHL, Cochrane and EMBASE. The review was restricted to publications that test outcomes of multidisciplinary collaboration in primary care in high-income countries. A conceptual model is used to structure the analysis.

**Results:**

Fifty-one studies comply with the selection criteria about collaboration in primary care. Approximately half of the 139 outcomes in these studies is non-significant. Studies among older patients, in particular, report non-significant outcomes (*p* < .05). By contrast, a higher proportion of significant results were found in studies that report on clinical outcomes.

**Conclusions and discussion:**

This review shows a large diversity in the types of collaboration in primary care; and also thus a large proportion of outcomes do not seem to be positively affected by collaboration. Both the characteristics of the structure of the collaboration and the collaboration processes themselves affect the outcomes. More research is necessary to understand the mechanism behind the success of collaboration, especially on the exact nature of collaboration and the context in which collaboration takes place.

## Introduction

Many countries stimulate collaboration between different disciplines in primary care and between primary care and other sectors [1]. There is a general agreement that collaboration is a way to establish a sustainable and affordable health care system. But to date the scientific evidence for this is ambiguous [1–3]. Nevertheless, policymakers, health care professionals and researchers strongly advocate the potential benefits such as increased efficiency-particularly regarding costs-more satisfaction for professionals allowing them to perform a greater variety of roles and better health outcomes for patients [4, 5].

Earlier research on collaboration does not always show these benefits [4, 6, 7]. Most of the studies conclude that, even now, little is known about the direct impact on outcomes of collaborative working in primary health care. This is in spite of the large body of studies on this topic. A key problem is that collaborative care models are examples of ‘complex interventions’ which consist of a number of separate elements. The particular elements that function as the ‘active ingredient’ can be difficult to identify [8].

There are often two main problems with studies on collaboration in primary health care that can lead to ambiguous results. The first problem is that most studies focus on one specific diagnosis or group of patients. This makes their results difficult to compare and as a result does not provide reliable evidence on how far general conclusions can be drawn from their outcomes. In addition, it also creates the risk of new and probably unnecessary separate ‘silos’ where people operate in isolation, such as care professionals organized and collaborating around one specific condition [9] and thus leaving no room for organizing and evaluating care in an holistic, integrated way [9, 10]. Moreover, focusing on a specific diagnosis or group of patients denies the increasing multi-morbidity in most western countries [11, 12], and this focus thus might not work for these more complex patients.

The second problem is that the actual collaboration, meaning the form and the activities carried out in collaboration, is poorly described. As a result, no replication, evidence synthesis and implementation in different situations can be carried out [8]. Therefore, it is hard to judge what actions in general are more successful or unsuccessful [13].

This review tries to overcome these problems by bringing together the studies on collaboration regardless of all their different patient populations, collaboration characteristics and activities. By doing so, and by identifying similarities between studies, a new light is shed on the outcomes of collaboration. To our knowledge, earlier reviews have never taken this approach. This conceptual model is based on the structure, process and outcomes model of Donabedian [14] and is adapted to collaboration in primary care. It is used to guide the research and analysis in this paper and to answer the main question: ‘What structures and processes of multidisciplinary collaboration in primary health care are reported in scientific literature and what conclusions can be drawn about its effect on patients’ and professionals’ outcomes?’

## Methods

### Search strategy

A Systematic Literature Review was performed to select and analyse scientific publications on collaboration in primary care. PubMed, CINAHL, Cochrane Library and EMBASE were searched for relevant studies in July 2014. We developed a Boolean search strategy for PubMed incorporating potential synonyms. The elements of our research question determined the search terms (MeSH). This search strategy was fine-tuned for the other databases. We searched under the four categories: (1) Multidisciplinary, (2) Collaboration, (3) Primary Health Care and (4) Outcomes. The search strategy was executed by combining all the terms within one type of search term to search for synonyms within (1), (2), (3) and (4)) together with a Boolean ‘OR’ operator. The four sets of terms were joined together with the ‘AND’ operator. All search terms for the electronic databases are available as a supplement.

### Criteria for including or excluding studies for this review

The inclusion criteria are:

- Language: English, Spanish, German and Dutch, as these are the languages we understand.
- Countries: 68 member states of the World Health Organization that fall into the category of high income countries in 2011.
- Methods: Only studies with quantitative data as primary outcomes had to be defined in quantitative measures. This makes it possible to compare the primary outcomes and judge them on their significance.
- Object of study: Does it describe an intervention with activities carried out in collaboration in primary care? Some form of communication between the disciplines involved in the care provided had to be part of the collaboration, while studies about the training of undergraduates within schools or universities were excluded.
- The article describes outcomes for patients, primary health care professionals or organizations. Studies that only reported on costs without reference to other outcomes were excluded, since this was not the main focus of our paper.

### Screening process

**Figure 1.**
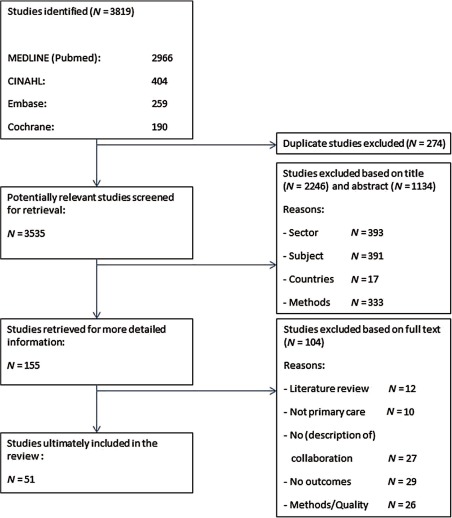
The screening process.

Figure 1 shows the results of the screening process. The search initially yielded 3535 unique references over the four databases. These were first assessed on the title and next on the abstract. If neither covered multidisciplinary collaboration, the article was excluded. The first 10% of the abstracts were reviewed by two reviewers (SMS and JH). Subsequently, the researchers resolved any disagreements by discussion and made the inclusion criteria more explicit. The remaining abstracts were reviewed by either one or the other. Next, the full text of 155 publications selected were reviewed independently by five reviewers (SMS, RB, DB, IP and JH). SMS read all publications and RB, DB, IP and JH read a part of all references. In this way, every publication was read by two reviewers. The inter-rater reliability kappa of the researchers giving exactly the same judgement is 0.73 (*p* < 0.05) which means the agreement is substantial. The few disagreements were resolved by discussion. In two cases, the full text of the publications was read by a third reviewer in order to resolve the disagreements that could not be solved by discussion.

The literature identified was dominated by case studies that did not report quantitative data and analysis and were therefore excluded. The same applies to studies where there is a main focus on another subject other than collaboration. We also excluded 12 reviews which were relevant studies but which had already been retrieved in our own search. This indicated that our search strategy was valid. Eventually 51 studies remained after duplicates were eliminated and studies were excluded based on title, abstract and full text.

### Quality assessment

We used the criteria of Cochrane Collaborations Effective Practice and Organization of Care (EPOC) [15] to assess the quality of the studies since the question posed by this research asks for more than just experimental study designs. We wished to investigate the effects of context on the effectiveness of an intervention. However, we also expanded the criteria with non-experimental designs, such as cohort studies and cross-sectional studies [16]. These studies were judged and scored on the quality of their methods. The quality rating of the studies included differed [high, moderate or low risk of bias (see Appendix, Table 2)]. A study was judged as having a low risk of bias if it met all seven criteria, a moderate risk of bias if it met four to six criteria and a high risk of bias if it met three criteria or fewer [15]. The designs that did not follow the EPOC criteria were judged as having a high risk of bias and, thus, a low quality, as these lack evidence when studying the effects of interventions. These studies with very low quality were excluded.

### The conceptual model

**Figure 2.**
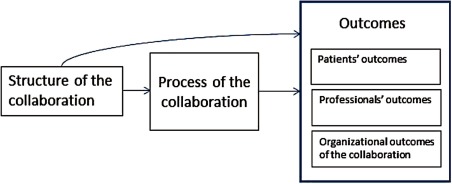
Conceptual model to analyse studies on collaboration in primary health care.

The studies found show similarities. These similarities were structured by the structure, process and outcomes model of Donabedian [14]. This model was applied to shed more light on the elements and effects of collaboration in primary care (Figure 2). By ‘structure’, we mean how the characteristics in which the collaboration is embedded were defined [14]. It includes the factors: (1) the team composition that is the presence of a primary care physician in the collaboration [17]; (2) the number of disciplines working together, that is the size of the collaboration; (3) the patient population described by patients’ age and patients’ condition; and (4) the number of sectors included in the collaboration. The process characteristics concern the way collaboration is actually shaped in practice that is the way in which care is delivered in collaboration, including the processes between different people [18, 19]. Three main types of collaboration processes have been discerned in the studies. The collaboration was shaped by the introduction of some kind of integrated, multidisciplinary care plan, by implementing multidisciplinary meetings and by the introduction of some kind of coordination of care or case management. In his model, Donabedian looks at ‘outcomes’ as the effects of health care on patients or populations, including changes in health status (clinical outcomes), behaviour or knowledge as well as patient satisfaction and health-related quality-of-life (patient-related outcomes) [14]. Other models and studies include outcomes such as organizational effectiveness, use of health services such as referrals, or the commitment or satisfaction of professionals [5]. In our review, we added such outcomes for organizations and for professionals, to the conceptual model. We grouped the outcome parameters used in the studies into four types: measures reported by patient such as health status, activities of daily living or patient satisfaction; clinical outcomes such as systolic blood pressure or HbA1c; the utilization of health care such as hospital admissions or use of emergency services (all outcomes for patients); and outcomes reported by professionals such as costs or job satisfaction (see Appendix). This review focused on outcomes for patients and professionals.

### Analysis

We conducted the analyses in two steps. First, the studies were coded and mapped according to the elements of our conceptual model: structure, process and outcomes (see Figure 1). Here, the process activities were limited to the three activities that were most commonly used. Those activities were: (1) a care plan for each individual developed by a multidisciplinary group of professionals; (2) multidisciplinary meetings; and (3) case management. Second, the outcomes reported in the publications were pooled. In doing so, this second analysis was performed on the level of the outcomes of the interventions, not on publication level. The 139 outcomes were coded as being statistically and positively significant (1) or not (0).This provided more detail on the outcomes of the studies, which would be missed if we had only reported on the publication level. Due to the, still small, number of outcomes, tests were only performed if the total set of the outcomes and its significant percentage was at least 10. We tested if the total of the different structural characteristics of the interventions are related to the significance of its outcomes. Likewise, we tested if the significance of the intervention is related to the different process activities of the intervention. The test we performed was a two-proportion *z*-test. It is used to determine whether the difference between two proportions is significant.

## Results

### Analysis 1: the characteristics of all 51 empirical studies

The largest share of studies in this review was conducted in the USA (*N* = 22) [17, 20–40], Canada (*N* = 8) [41–48] and the UK (*N* = 7) [49–55]. Five studies investigated the Netherlands [56–60], two Sweden [61, 62], and one study each was from Belgium [63], New Zealand [64], France [65], Spain [66], Israel [67], Australia [68] and Puerto Rico [69]. The largest share of study designs is randomized controlled trials (*N* = 18) [17, 27, 29, 31–34, 38, 39, 42, 43, 50, 54–56, 60, 64, 69] and controlled clinical trials (*N* = 5) [25, 26, 45, 48, 61]. All studies were published between 1993 and July 2014. The length of the intervention differed between the studies and was not always described. However, the duration of the research and the time between the first and last measurement was described. This was on average 14 months, ranging from 3 to 48 months. The conditions of the patients included in the studies and collaborations are: chronic conditions, 19 studies of which 7 related to diabetes and 3 to uncontrollable hypertension; multiple mental conditions comprising 10 studies of which 7 were about depression; and 22 studies in which conditions are not specified. The key characteristics of the studies included in this review are summarized in Appendix.

Table 1 compares the structural characteristics with the process activities of the 51 studies selected. Patients individual care plans and multidisciplinary meetings were implemented to improve the collaboration processes in a majority of the studies, 23 and 20 studies, respectively.

Care coordination by a case manager is the third most frequently found activity, occurring in 13 studies. A GP/physician was involved in the collaboration in the majority of the interventions. Most (60%) of the interventions are medium or large collaborations involving four or more different disciplines rather than small collaborations. Interventions that included two or three different collaborating disciplines use individual care plans more frequently. However, interventions where four or more different disciplines are involved more frequently collaborate through multidisciplinary meetings. A relatively large share of interventions, occurring in 18 papers, focused on an older patient population.

The majority of these interventions work with individual care plans. Studies about other age categories, some non-specific, describe interventions more frequently with multidisciplinary meetings.

The interventions are often targeted at patients with chronic, or multiple chronic, conditions (19 studies), or at patients with mental, or multiple mental illnesses (10 studies). Eight studies tested interventions that use individual care plans for patients with one or more chronic conditions. The studies describe not only interventions involving collaborations within primary care itself, but also half of them (25 studies) involve interventions between primary care and one or more other sectors such as mental health or secondary care. Interventions about collaborations within primary care mostly use individual care plans. However, interventions involving collaboration with different sectors such as mental health or secondary care are more frequently carried out through multidisciplinary meetings.

The structure and process characteristics of the interventions are related to the outcome parameters used in the studies in Table 2. Most studies report the clinical outcomes resulting from the interventions (25 studies) or outcomes related to the patient (23 studies). Almost all studies report more than just one type of outcome parameter. Only two studies show outcomes from the collaborations where there was no physician involved. The following structural characteristics of the interventions test the outcomes related to the patient more frequently: collaborations with four or more different disciplines; older people; patients with a mental illness; patients with other conditions or where their conditions were not specified; and collaborations in more than one sector. The studies which most often report clinical outcomes were: interventions involving small collaborations, interventions that included patients with chronic or multiple chronic conditions and collaborations within primary care. Studies about multidisciplinary meetings most frequently report outcomes related to the patient, whereas the four different categories of outcomes are spread over the other activities.

### Analysis 2: the 139 outcomes reported in the 51 studies

One hundred and thirty-nine different outcomes were reported in the 51 studies. Taking these 139 outcomes as the unit of analysis provides an opportunity to estimate what evidence is present to suggest a successful collaboration and to what extent the structures and processes of the collaborations are related to success. Of the studies in this review, 16 studies did not show any significant outcomes. Table 3 shows the four types of outcome parameters and the proportion of statistically significant outcomes related to the structure or process characteristics of the interventions. The largest share of the 139 reported outcomes is clinical (50 outcomes). These were also most frequently found to be positive and significant (54%).

The last column of Table 3 shows the results of the two-proportion *z*-tests. When tested there was no significant difference between interventions with or without a physician involved in the collaboration. Neither did we find that medium or large interventions, in terms of number of disciplines involved, coincide more often with positive and statistically significant effects for each type of outcome. In the case of smaller collaborations, the only category that was most likely to be significant is the proportion of clinical outcomes. However, these specific outcomes were not tested because of the small number. When comparing non-specific age groups to older people, there is a significant difference in the outcomes. Non-specific age groups more often show a positive significant outcome (*p* < .05). It is only for the outcome category ‘use of health services’ that a higher proportion of positive and significant outcomes was reported for interventions targeted at older people. There is no significant difference in outcomes between the patients’ conditions. Table 3 shows that interventions for patients with physical or multiple physical chronic conditions report more significant clinical outcomes (not tested). It seems that inter-sectoral interventions outperform interventions within primary health care. However, this is not significant.

When looking at the collaborative activities, that is the type of collaboration process, care coordination interventions have the highest proportion of significant effects on the four different outcomes. In general, if interventions report using multidisciplinary meetings, they show relatively more outcomes and significant outcomes in those outcomes related to a professional. Individual care plans most often report positive outcomes on clinical outcomes.

## Discussion

The objective of this study was to perform a review of scientific papers that present empirical results about multidisciplinary collaborations in primary health care and their effect on patients’ and professionals’ outcomes. We believe this review is the first in which empirical studies on collaboration in primary care and its outcomes, and not limited to specific patient groups or diseases or the type of collaboration, are compared and analysed.

The studies found in this review reported on the structure and outcomes. However, the processes were often poorly described and monitored. As a consequence, it remains difficult to investigate the expectation that processes could be as important as structures for outcomes of primary care collaborations. However, of the process activities, the three most reported and clearly described activities were extracted from the studies and tested in this review. Because of a small number of studies and outcomes, the three components of the conceptual model could not be tested simultaneously.

Most of the 51 studies analysed focused primarily on the outcomes for patients. A few studies reported outcomes for professionals. The underlying reason for this might be that outcomes for patients are considered to be the most important and therefore these are the most researched. However, it remains relevant to investigate too if collaborations that show better outcomes for professionals also lead to better care for patients. In future research this aspect should be taken into account.

The 51 papers and the 139 outcomes reported in this review show that collaboration in primary care equally does, and does not, result in positive and significant outcomes. The proportion of significant outcomes in the 51 papers is lower than 50%. We believe this is a significant insight given the common belief that collaboration in primary care will generally result in greater efficiency-including cost efficiency, greater professional satisfaction due to task enrichment and better health outcomes for patients. We find that this is *not* supported by the collaborations reported in the studies and analysed in this review. We believe there are possible reasons why the results of collaboration in our review are disappointing:

- Collaboration is not leading to better outcomes, at least not all types of collaboration interventions lead to better outcomes;
- The intervention involved in the collaboration was not implemented properly in some of the studies. For instance, a protocol was introduced but not all professionals adhered to it.
- The research project was not able to identify the effect on outcome.

All three reasons are plausible given our results. It might be that some interventions are less successful, for instance for older people. Also, the idea of the collaboration may not sometimes have been implemented properly. However, the last reason seems the most plausible in our review. First, the quality of the studies might result in a bias towards the opportunity to find significant outcomes. By using the EPOC tool, the risk of bias in the studies was screened out, leading to exclusion of studies of very low quality (26 based on full text). However, there remained 21 studies of low quality included in this review. This low quality could have influenced the results and could lead to them becoming less significant. Second, the sensitivity of the outcomes we measured limits the opportunity to find significant outcomes. Some outcomes are harder to improve by investing in collaboration than others. Studies that do show an improvement mostly concern clinical outcomes. This is probably related to the fact that collaborations which focused on specific diseases have better conditions to show clinical outcomes. In general, the clinical effects in one specific patient group can be better quantified and are more easily achieved in a shorter time. Finally, the follow-up in the measurements might be too short a period of time for outcomes to be realized. The studies in this review varied in their period of measurement from three months to four years. We did not account for this as it was beyond the scope of this review. However, the review of Kruis [7] showed that in the short term (three to 12 months) almost all outcomes for patients with anxiety and depression improved, but in the long term (>12 months) the effect remained unclear. Outcomes require some time but this does not automatically hold true for the longer term. The three reasons should be taken into account in further attempts to implement collaboration and in the monitoring of research.

The large number of studies in this review that address the involvement of primary care physicians reflect perhaps the dominant and crucial position that physicians have in many health care systems. Some characteristics of the structure of collaborations show higher positive significant outcomes, such as the involvement of a physician in the collaboration compared to no involvement of a physician (not significant). The paper of Poulton [53] showed that the size and effectiveness of the team in health care collaboration are not related. Our review supports this finding. Although other studies [70] reveal that it is harder to collaborate when more disciplines are involved, we found the opposite: the proportion of significant clinical outcomes is higher if collaborations involve more disciplines. Furthermore, we found that interventions for patients with chronic conditions show more significant clinical outcomes and use of health services if multidisciplinary collaborations are involved. This support claims that for this group of patients gains from collaboration can be achieved [71–73].

It is suspected that collaborating between sectors can have more language or payment barriers [53]. This is not found to be the case in this review. Collaborations between primary care and providers from other sectors show no significant difference from other collaborations. However, regarding the characteristics of the structure, only the non-specific age groups show a significant difference than with older people. Collaborations that are directed towards older patients show less positive significant clinical and patient-related outcomes and did not offer any extra benefit for professionals. This might be understood by the fact that older people more often have multiple chronic diseases and are more fragile. As a consequence, improving collaboration for this group of patients, though important, is difficult to establish. This is probably why it is harder, compared to other groups, to improve the clinical and patient-related outcomes in general, and specifically, through better collaboration. This might also result in lower satisfaction for the professionals who are involved in such collaboration projects. This result challenges the assumption that more collaboration is the best way to improve the health care services for older patients.

When it comes to the process of collaboration, it appears most beneficial to implement some form of care coordination, especially when trying to improve clinical and patient-related outcomes. However, the difference in outcomes between the activities is small. Collaborations that include multidisciplinary meetings more often than the other activities show a higher proportion of significant outcomes for professionals. This may indicate that these meetings help professionals simply get to know each other and thus stimulate team building [18].

## Limitations

Interventions to improve collaboration show a large variation in the structure of the collaboration, the actual collaboration process that is improved and in the implementation of the interventions. However, grouping the interventions by their characteristics of structure and process reveals significant patterns.

Nevertheless, the results may be subject to both a publication and study selection bias. Publication bias may occur as studies will be more likely to get published when the outcomes show an effect. This could overestimate the proportion of significant outcomes. At the same time, we find that 16 of the 51 studies in this review did not report any positive significant effect. Study selection bias occurs as this review is limited to studies using quantitative data from high-income countries. As a result, general conclusions should not be applied to other, middle- or low-income countries. Our selection of databases in health care could also have resulted in a study selection bias. Still, these databases are the most common and suitable to retrieve studies on collaborations and outcomes in health care. This was reflected by the fact that all relevant studies incorporated in previous reviews were also found in our search strategy and in the databases we used. Moreover, the selection process included a selection on outcomes, which excluded some studies such as those on costs alone.

The study selection was first undertaken on title and later on abstract and on full text. The selection on title might have excluded studies with a title giving no clue about the objective of the study. However, when in doubt the study's abstract was screened.

Our data set is essentially cross-sectional and the numbers involved are rather small. We tried to overcome this shortcoming by testing on the number of outcomes (*N* = 139), instead of focusing on the number of studies (*N* = 51). Still, this number is too small for multivariate analysis. Therefore, it is not possible to draw causal inferences on the effect of the type of study and the outcomes involved. To overcome problems with multiple testing, a two-proportion *z*-test is used. By reducing the analysis to separate structures and activities, the variation is made as small as possible, and comparisons are made on the level of these separate elements. For example, the three most reported activities were chosen for the analysis. Unfortunately, the intensity and quality of these actions could not be taken into account because of a poor description.

Much research on collaboration has been carried out, often focusing on small parts of a collaboration [25, 74, 75]. But in this review, all the different studies are combined and compared, for example collaborative initiatives for type 2 diabetes patients compared to studies on patients with mental health. This should not be a problem since the collaborative activities carried out are often the same, for example a multidisciplinary meeting or care coordination. Moreover, the strength of this review lies in focusing on outcomes rather than individual studies.

In conclusion we argue that:

- Until now collaboration in many studies does not live up to its expectations with less than 50% of the outcomes positive and significant. However, this might be the problem of the quality of the studies, the expectation riding on some outcomes and the follow-up time;
- A higher proportion of significant results of collaboration was found in studies that report on clinical outcomes;
- Many studies suggest collaboration for older people is unsuccessful.

The important implications for daily practice are that implementation of a collaboration resulting in outcomes takes time and effort, especially in the long run. It is important to monitor the process and special attention is required for older patients.

## Figures and Tables

**Table 1.**
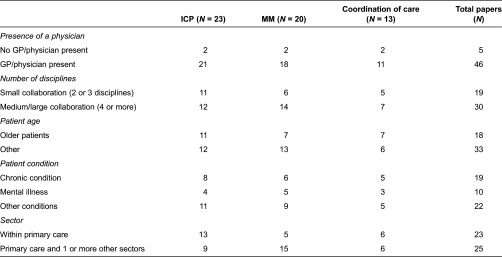
Structure and process characteristics of the interventions (*N* = 51 studies)

ICP, individual care plan; MM, multidisciplinary meetings

**Table 2.**
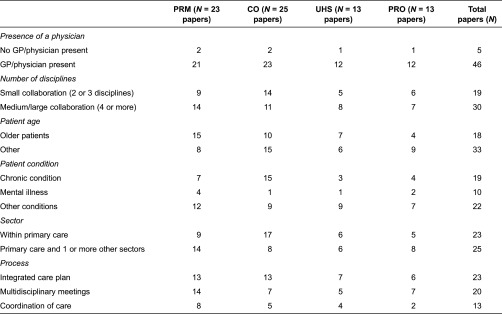
Structure and process characteristics of the interventions related to the type of outcome parameters used in the studies (*N* = 51 studies)

PRM, patient-reported outcomes; CO, clinical outcomes; UHS, use of health services; PRO, professional-reported outcomes

**Table 3.**
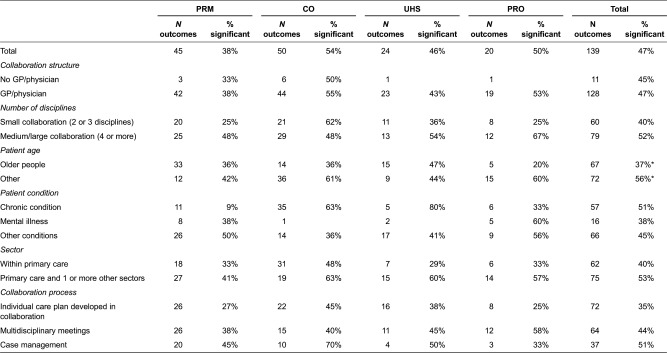
% significant positive effects of structure and process characteristics of collaboration on outcomes (*N* = 139 outcomes)

PRM, patient-reported outcomes; CO, clinical outcomes; UHS, use of health services; PRO, professional-reported outcomes

*p < .05 (within each characteristic is tested with a two-proportion *z*-test)

**Table 1.**
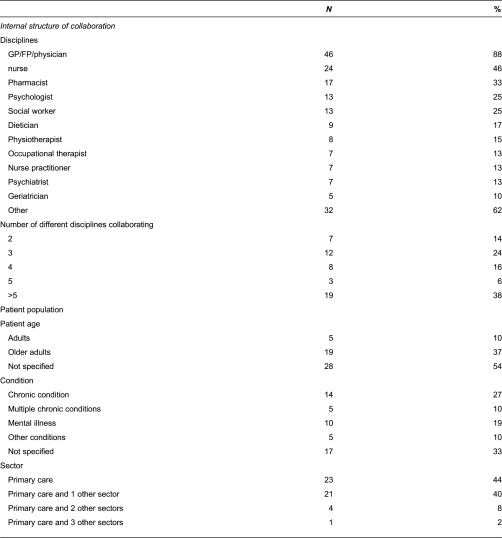

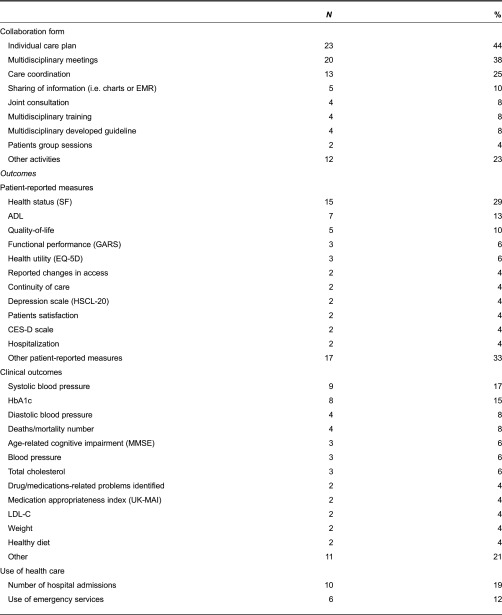

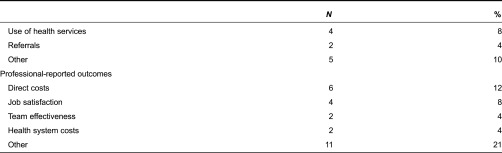
Description of the selected studies (*N* = 51 papers)

ADL, activities of daily living; GARS, Groningen Activity Restriction Scale; CES-D Scale, Centre for Epidemiologic Studies-Depression Scale; MMSE, mini mental state examination; LDL-C, low density lipid cholesterol

**Table 2.**
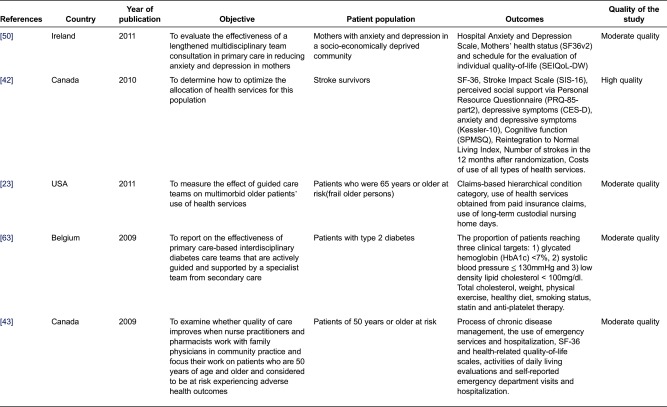

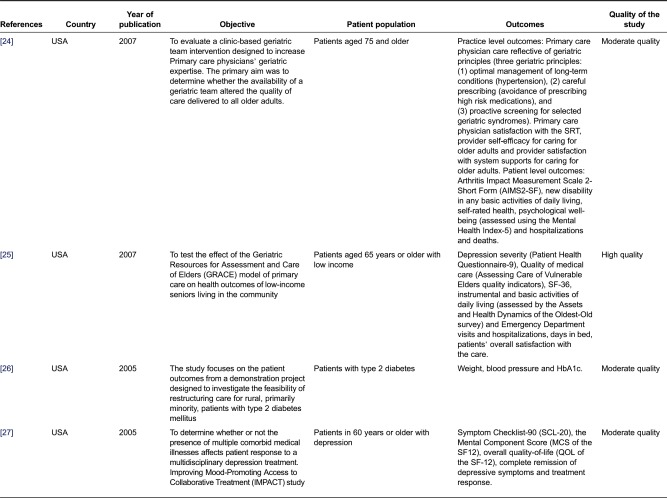

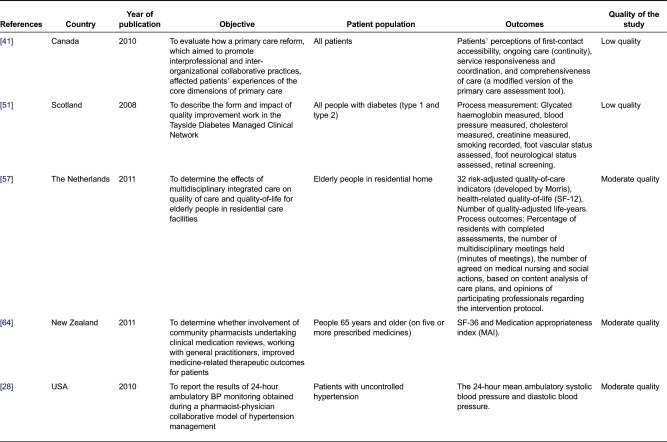

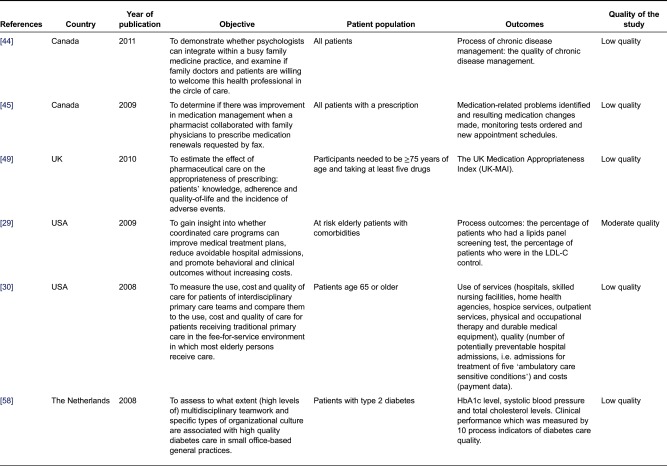

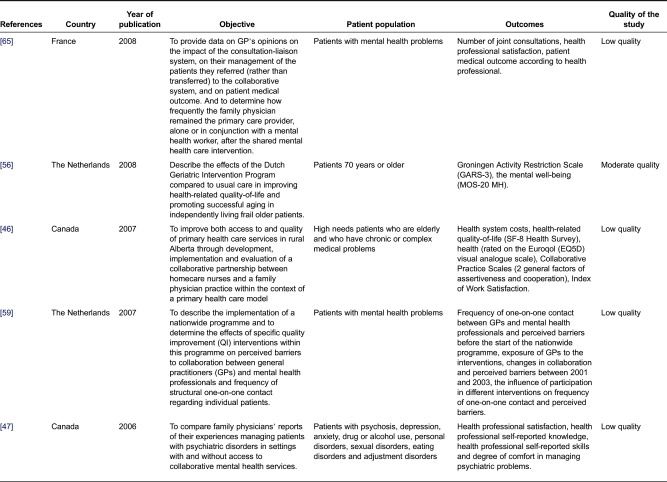

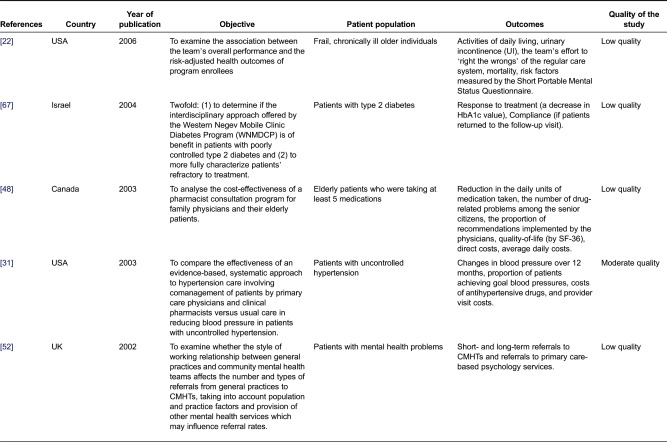

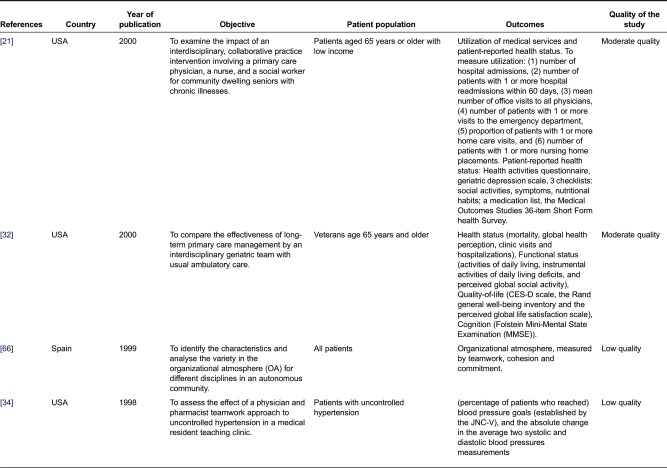

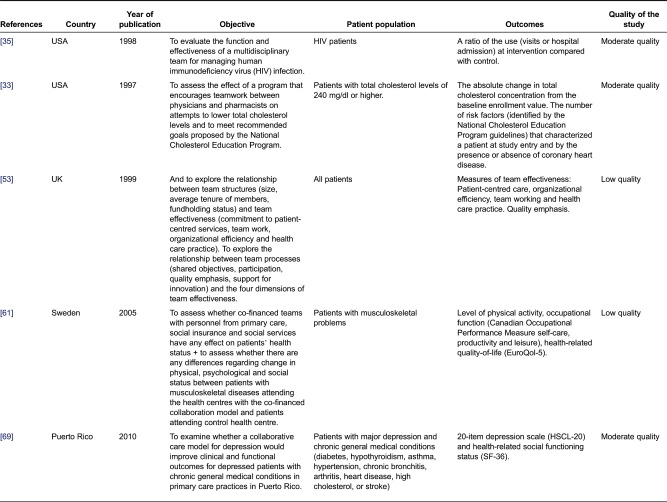

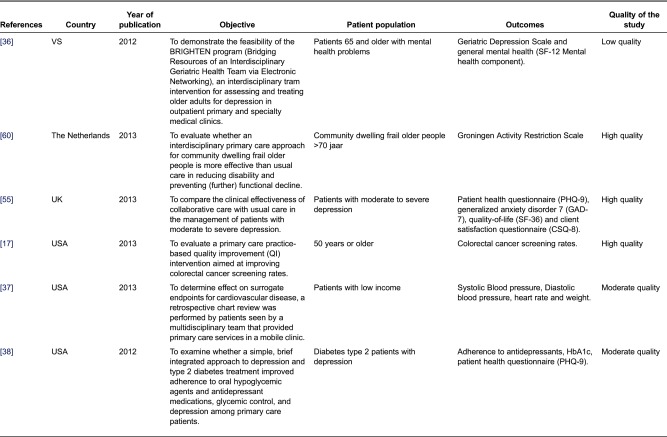

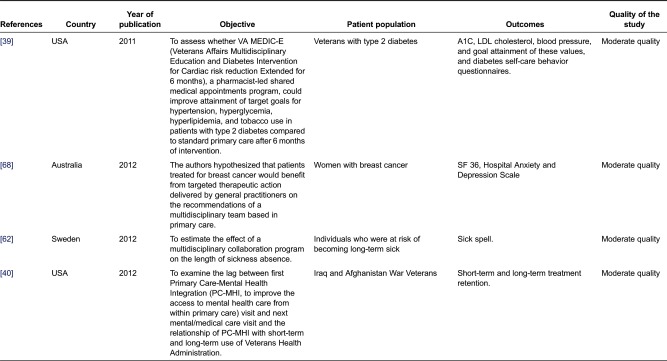
Objective, outcomes and quality of the selected studies
